# Low-dose tacrolimus combined with donor-derived mesenchymal stem cells after renal transplantation: a prospective, non-randomized study

**DOI:** 10.18632/oncotarget.7725

**Published:** 2016-02-25

**Authors:** Guang-hui Pan, Zheng Chen, Lu Xu, Jing-hui Zhu, Peng Xiang, Jun-jie Ma, Yan-wen Peng, Guang-hui Li, Xiao-yong Chen, Jia-li Fang, Yu-he Guo, Lei Zhang, Long-shan Liu

**Affiliations:** ^1^ The Transplantation Centre, The Second Affiliated Hospital, Guangzhou Medical University, Guangzhou, China; ^2^ Center for Stem Cell Biology and Tissue Engineering, SunYat-sen University, Guangzhou, Guangdong, China; ^3^ Laboratory of General Surgery, The First Affiliated Hospital, Sun Yat-sen University, Guangzhou, China

**Keywords:** acute rejection, calcineurin inhibitors, graft survival, mesenchymal stem cells (MSCs), nephrotoxicity, Pathology Section

## Abstract

Calcineurin inhibitors, including tacrolimus, are largely responsible for advances in allotransplantation. However, the nephrotoxicity associated with these immunosuppressants impairs patients' long-term survival after renal allograft. Therefore, novel regimens that minimize or even eliminate calcineurin inhibitors could improve transplantation outcomes. In this pilot study, we investigated the use of low-dose tacrolimus in combination with mesenchymal stem cells (MSCs), which are immunosuppressive and prolong allograft survival in experimental organ transplant models. Donor-derived, bone marrow MSCs combined with a sparing dose of tacrolimus (0.04-0.05 mg/kg/day) were administered to 16 *de novo* living-related kidney transplant recipients; 16 other patients received a standard dose of tacrolimus (0.07-0.08 mg/kg/day). The safety of MSC infusion, acute rejection, graft function, graft survival, and patient survival were evaluated over ≥24 months following kidney transplantation. All patients survived and had stable renal function at the 24 month follow-up. The combination of low-dose tacrolimus and MSCs was as effective as standard dose tacrolimus in maintaining graft survival at least 2 years after transplantation. In addition, both groups had similar urea, urine protein, urinary RBC, urinary WBC, 24-h urine protein, and creatinine clearance rates from 7 days to 24 months after transplantation. Furthermore, no differences in the proportion of lymphocytes, CD19, CD3, CD34, CD38, and natural killer cells were detected between the control and experimental groups. None of the MSC recipients experienced immediate or long-term toxicity from the treatment. This preliminary data suggests that the addition of MSCs permits the use of lower dosages of nephrotoxic calcineurin inhibitors following renal transplantation.

## INTRODUCTION

Kidney transplantation remains the most effective therapy for patients with end-stage renal disease. The major barrier to renal transplantation is acute and chronic rejection of engrafted kidneys by the recipient's immune system, hence the need for immunosuppressants. Conventional immunosuppressive drugs are generally used in transplant patients and are effective in reducing acute rejection and improving short-term outcomes. However, long-term graft survival remains a major problem after renal transplantation.

Tacrolimus is a calcineurin inhibitor that binds FKBP12, suppressing nuclear factor of activated T cells (NFAT) transcription of cytokines and, therefore, T cell function [1]. It is highly effective in preventing acute rejection and improving short-term survival in renal allograft recipients [2]. However, its adverse effects, such as nephrotoxicity, diabetogenicity, and neurotoxicity, constrain its long-term utility [3–5]. Thus, the elimination or minimization of calcineurin inhibitors is required to attain further improved outcomes in kidney transplantation [6]. It is necessary to develop novel immunosuppressive regimens that maximize the beneficial effects of transplantation tolerance without the aforementioned adverse effects.

Mesenchymal stem cells (MSCs) are multipotent progenitor cells that can be induced to undergo rapid proliferation and differentiation into multiple cell lineages [7, 8]. In addition, a myriad of studies have shown that MSCs activate the proliferation of T cells and natural killer (NK) cells, and decrease the maturation and function of antigen-presenting cells, such as dendritic cells. The immunosuppressive capacity of MSCs makes them a potentially important therapeutic target in transplantation as they may inhibit allograft rejection and induce transplant tolerance. MSCs have been used to enhance hematopoietic stem cell engraftment and to treat graft-*versus*-host disease (GVHD) and autoimmune disease [9-11]. In animal transplantation models, MSCs significantly prolonged skin and cardiac allograft survival [12-14]. Crop et al. [15] also found that infusion of donor MSCs could significantly suppress the proliferation of alloactivated T-cell subsets. These findings suggest that donor-derived MSCs may facilitate the induction of transplant tolerance, thus suppressing renal allograft rejection. Recently, Tan et al. [16] reported that the use of autologous MSCs resulted in a lower incidence of acute rejection, decreased risk of opportunistic infection, and better estimated renal function at 1 year among patients undergoing renal transplantation.

In a pilot study of 12 patients, we had previously shown that donor-derived MSCs combined with low-dose tacrolimus prevented acute rejection after renal transplantation [17]. The findings presented here are a continuation of that pilot study. In addition to the original 12 patients, 20 new patients were enrolled in this study. Sixteen patients were inoculated with MSCs and treated with low-dose tacrolimus (approximately 60% of the regular dose), while the other 16 received the standard dosage of tacrolimus. We show here that the combination of MSCs with low-dose tacrolimus was as effective as standard dose tacrolimus in preventing acute renal rejection and in maintaining graft survival at least 2 y after transplantation.

## RESULTS

### Baseline characteristics of all participants

As shown in Table 1, the two groups had similar baseline characteristics, including age (mean = 29.97 y, SD = 8.34, range: 16-51 y), sex, HLA mismatching (50% of patients had one), estimated glomerular filtration rate (eGFR, mean = 5.16 mL/min, SD = 3.06 mL/min, range: 1.88-18.60 mL/min), serum creatinine (mean = 1211.84 μmol/L, SD = 412.50 μmol/L, range: 369-2657 μmol/L), urea (mean = 24.89 mmol/L, SD = 11.25 mmol/L, range: 9.53-52.96 mmol/L), urine protein (mean = 2.08 q/I, SD = 0.99 q/I, range: 0.3-3.0 q/I), urinary RBC (mean = 44.85 count/μL, SD = 61.22 count/μL, range: 0-246 count/μL), urinary WBC (mean = 107.59 count/μL, SD = 441.75 count/μL, range: 0-2391 count/μL), lymphocytes (LN) percentage (mean = 24.51%, SD = 6.20%, range: 3.5-34.3%), LN count (mean = 1.5×10^9^/L, SD = 0.39×10^9^/L, range: 0.75-2.33×10^9^/L), CD19 (mean = 11.72%, SD = 4.85%, range: 3.3-21.7%), CD3 (mean = 72.33%, SD = 9.97%, range: 55.1-90.4%), CD34 (mean = 40.52%, SD = 8.1%, range: 22.7-56.7%), CD38 (mean = 28.77%, SD = 8.5%, range: 13.7-45.6%), and NK levels (mean = 7.6%, SD = 3.01%, range: 2.8-13.31%). The follow-up duration of the two groups was also similar (mean = 28.14 months, SD = 10.8 months, range: 14-49.8 months). However, the experimental group had higher levels of urinary RBCs than the control group. In addition, there was no difference in donors' characteristics between the two groups, including age (*P* = 0.183), gender (*P* = 0.473) and creatinine levels (*P* = 0.057).

**Table 1:** Baseline characteristics of the 32 patients

	Control (*n* = 16)	Experimental (*n* = 16)	*P*-value
Age (y)	29.31 (9.48)	30.63 (7.27)	0.664
Sex			
Males	11 (68.8)	15 (93.8)	0.172
HLA mismatching			0.325
1	8 (50.0)	8 (50.0)	
2	4 (25.0)	7 (43.8)	
3	4 (25.0)	1 (6.2)	
eGFR (mL/min)	5.29 (3.82)	5.02 (2.19)	0.812
Scr (μmol/L)	1215.19 (498.34)	1208.50 (321.40)	0.964
Urea (mmol/L)	23.93 (15.17, 36.56)	20.94 (16.88, 25.31)	0.468
Urine protein (q/L)^1^	1.25 (1.00, 3.00)	3.00 (2.00, 3.00)	0.201
Urinary RBC (count/μL)^1^	6.00 (2.00, 26.00)	66.00 (5.00, 119.00)	0.041*
Urinary WBC (count/μL)^1^	8.00 (3.00, 38.00)	11.00 (4.00, 23.00)	0.747
Percentage of LN (%)	25.86 (5.83)	23.17 (6.44)	0.225
LN count (10^9^/L)	1.58 (0.42)	1.42 (0.36)	0.269
CD19 (%)	12.32 (4.62)	11.12 (5.16)	0.494
CD3 (%)	73.66 (9.09)	71.01 (10.91)	0.462
CD34 (%)	39.77 (8.01)	41.27 (8.39)	0.609
CD38 (%)^1^	30.94 (9.38)	26.73 (7.28)	0.172
NK (%)	7.20 (6.00, 10.67)	7 (5.04, 8.37)	0.445
Follow-up duration (months)	23.87 (18.68, 33.03)	30.07 (19.68, 33.50)	0.468
Age of donors	45.56 (12.98)	50.56 (6.85)	0.183
Male gender of donors	8 (50.0%)	5 (31.3%)	0.473
Creatinine of donors	71.06 (8.96)	77.69 (9.97)	0.057

eGFR, estimated glomerular filtration rate; Scr, serum creatinine; RBC, red blood cell; WBC, white blood cell; LN, lymphocytes; NK, natural killer

Continuous data were presented as mean (standard deviation) or median (interquartile range) and tested by the independent t-test or Mann-Whitney U test.

HLA mismatching was expressed as n (%) and tested by Fisher's exact test.

* Indicates significant difference between the two groups, *P* <0.05.

1 Some missing data were found.

### Levels of urea, urine protein, urinary RBC, urinary WBC, 24-h urine protein and creatinine clearance rate (Ccr) over time

Both groups had similar changes of urea, urine protein, urinary RBC, urinary WBC, 24-h urine protein levels and Ccr levels over the study period (all *P*_group effect_ > 0.05; Table 2). In both groups, the levels of urea dropped in the first 3 months following the surgery and were maintained thereafter. Likewise, the levels of urine protein dropped after the surgery, with a maximal decrease at 1 month that was maintained. Whereas the urine RBC levels increased at day 7 following the surgery, they sharply decreased at one month after the surgery, and no significant changes were subsequently observed. The urine WBC levels dropped significantly within 3 months after the surgery and then stabilized. However, no significant changes in the concentrations of 24-h urine protein and Ccr were observed (Table 2).

**Table 2:** Comparisons of urea, urine protein, urinary RBC, urinary WBC, 24-h urine protein, and Ccr between the control (n = 16) and experimental (n = 16) groups

	Group	Baseline		7 days	1 M		3 M	6 M		12 M		24 M
Urea (mmol/L)^1^	Control	27.23 (12.75)	13.96 (10.38)^†^			9.83 (7.63)^†^	6.58 (1.94)^†,§^		6.25 (1.39)^†,§^		5.96 (1.46)^†,§^	6.59 (2.07)^†,§^
	Experimental	22.54 (9.34)	13.25 (5.19)^†^			8.5 (3.07)^†^	6.24 (1.54)^†,§^		6.67 (1.89)^†,§^		6.07 (1.76)^†,§^	5.71 (1.75)^†,§^
Urine protein (q/l)^2^	Control	1.25 (1, 3)	0.3 (0.15, 0.85)^†^			0 (0, 0)^†,§^	0 (0, 0)^†,§^		0 (0, 0.1)^†,§^		0 (0, 0)^†,§^	0 (0, 0)^†,§^
	Experimental	3 (2, 3)	0.25 (0.1, 0.35)^†^			0 (0, 0.2)^†,§^	0 (0, 0)^†,§^		0 (0, 0)^†,§^		0 (0, 0)^†,§^	0 (0, 0)^†,§^
Urinary RBC (count/μL)^2^	Control	6 (2, 26)	96.5 (39, 318.5)^†^			1.5 (0.5, 5.5)^†,§^	0.2 (0, 2)^†,§^		1 (0, 2)^†,§^		0 (0, 7) ^†,§^	1 (0, 2)^†,§^
	Experimental	66 (5, 119)	80.5 (44, 590)^†^			3 (1.2, 7.5)^†,§^	0 (0, 3.5)^†,§^		0.2 (0, 2.5)^†,§^		0 (0, 2.5)^†,§^	0.5 (0, 6)^†,§^
Urinary WBC (count/μL)^2^	Control	8 (3, 38)	12 (7.5, 38)			4.5 (2.5, 13.5)^§^	0.83 (0, 2)^†,§,¶^		1.5 (0, 2.5)^†,§,¶^		0 (0, 1.5)^†,§,¶^	0 (0, 0.66)^†,§,¶^
	Experimental	11 (4, 23)	13 (5.5, 17.5)			5 (2, 10)^§^	0.93 (0, 2)^†,§,¶^		0 (0, 1.5)^†,§,¶^		0 (0, 0)^†,§,¶^	0 (0, 1)^†,§,¶^
24-h urine protein (q/L)^1^	Control	ND	ND			216.28 (158.73)	176.86 (69.73)		136.71 (43.54)		144.11 (49.49)	ND
	Experimental	ND	ND			171.26 (84.43)	171.36 (134.51)		173.58 (179.37)		125.75 (75.11)	ND
Ccr (ml/L)^2^	Control	ND	ND			82.67 (51.25, 92.44)	69.29 (59.09, 86.18)		74.02 (67.26, 80.86)		77.9 (66.02, 91.75)	ND
	Experimental	ND	ND			63.54 (55.35, 78.27)	66.79 (61.25, 75.54)		68.75 (63.65, 80.52)		68.25 (61.6, 87.09)	ND

RBC, red blood cell; WBC, white blood cell; ND, not determined; Ccr, creatinine clearance rate.

1 Data were presented as mean (standard deviation) and tested using the linear mixed model.

2 Data were presented as median (interquartile range) and tested using the Friedman test for time effect and Mann-Whitney U test for group effect.

† significantly different from the baseline value, *P* < 0.003.

§ significantly different from Day 7, *P* < 0.003.

¶ significantly different from 1 month, *P* < 0.003.

### Changes in LN percentage, LN count, CD19, CD3, CD34, CD38 and NK cells over the study period

A significant group effect was found for CD38 (*P* = 0.010) and NK (*P* = 0.007); the percentage of CD38 and NK cells was higher in control group than in the experimental group. The percentages of both CD38 and NK cells in the control group were higher than those in the experimental. The LN percentage dropped after the surgery, and increased to the baseline level in both groups (*P* < 0.003). A significantly higher LN count was found at 3 months as well as 6 months as compared to day 7 (*P* < 0.003). Conversely, the proportion of CD19 cells increased, peaking at day 7 and then decreased gradually. The proportion of CD3 cells dropped at the same time and then fluctuated between 69% and 78% thereafter. The lowest percentages of CD34 and CD38 were detected at day 7; they increased at 1 month and were sustained thereafter. At 1 month, the CD34 percentage was significantly lower than the baseline value (*P* < 0.003). Finally, a significant reduction in the proportion of NK cells was found at day 7 (*P* < 0.003), and the values detected at other time points were similar with those observed at baseline (Table 3).

**Table 3:** Comparisons of percentage of lymphocytes, CD19, CD3, CD34, CD38, and natural killer cell between the control (n = 16) and experimental (n = 16) groups

Variables	Group	Baseline	7 days		1 M		3 M	6 M		12 M		24 M
Percentage of LN (%)^1^	Control	25.86 (5.83)		10.87 (5.49)^†^		15.91 (8.01)^†^	19.07 (6.31)		20.24 (6.79)		17.65 (5.54)	22.66 (5.20)^§^
	Experimental	23.17 (6.44)		16.23 (5.88)^†^		18.68 (8.33)^†^	17.91 (7.43)		18.76 (5.21)		21.33 (7.01)	25.02 (13.21)^§^
LN count (10^9^/L)^2^	Control	1.64 (1.22, 1.81)		1.00 (0.51, 1.27)		1.21 (1.00, 1.71)	1.72 (1.17, 16.59)^§^		1.40 (1.20, 10.35)^§^		1.15 (1.00, 1.40)	1.38 (1.36, 1.80)
	Experimental	1.42 (1.17, 1.68)		1.41 (0.70, 1.72)		1.41 (1.01, 1.72)	1.46 (1.03, 13.92)^§^		1.56 (1.15, 8.44)^§^		1.27 (1.13, 2.21)	1.47 (1.35, 3.35)
CD19 (%)^1^	Control	12.32 (4.62)		39.29 (17.13)^†^		17.7 (11.20)^§^	15.57 (8.71)^§^		12.58 (6.73)^§^		11 (4.19)^§^	12.18 (6.19)^§^
	Experimental	11.12 (5.16)		32.54 (19.55)^†^		20.13 (10.34)^§^	17.88 (12.58)^§^		12.57 (8.46)^§^		11.18 (5.48)^§^	13.79 (6.67)^§^
CD3 (%)^1^	Control	73.66 (9.09)		47.88 (18.57)^†^		69.2 (11.62)^§^	74.11 (7.75)^§^		77.1 (11.20)^§^		78.24 (7.19)^§^	73.38 (9.46)^§^
	Experimental	71.01 (10.91)		53.02 (20.61)^†^		71.28 (11.90)^§^	72.75 (11.58)^§^		72.99 (15.06)^§^		75.03 (9.32)^§^	71.91 (9.96)^§^
CD34 (%)^1^	Control	39.77 (8.01)		21.95 (9.16)^†^		31.62 (8.94)^†^	30.76 (7.88)^†^		32.66 (11.10)^†^		32.68 (9.98)^†^	30.29 (7.89)^†^
	Experimental	41.27 (8.39)		27.97 (12.28)^†^		30.99 (11.15)^†^	31.25 (9.74)^†^		29.86 (8.83)^†^		31.37 (6.25)^†^	30.08 (7.84)^†^
CD38 (%)^1^	Control	30.94 (9.38)		22.6 (9.26)		37.44 (10.36)^§^	36.48 (8.86)^§^		35.1 (9.50)^§^		35.63 (12.07)^§^	33.06 (6.20)
	Experimental	26.73 (7.28)		23.71 (6.6)		32.22 (8)^§^	31.44 (7.68)^§^		33.55 (10.49)^§^		32.92 (8.29)^§^	28.99 (4.68)
Natural killer cell (%)^1^	Control	8.06 (3.01)		4.46 (2.56)^†^		5.51 (3.66)	6.65 (3.43)		5.92 (4.42)		5.86 (3.85)	7.83 (4.64)
	Experimental	7.13 (3.03)		2.93 (2.41)^†^		4.37 (3.41)	4.76 (3.09)		6.02 (4.1)		4.94 (2.68)	6.55 (4.74)

LN, lymphocytes

1 Data were presented as mean (standard deviation) and tested using thr linear mixed model.

2 Data were presented as median (interquartile range) and tested using the Friedman test for time effect and Mann-Whitney U test for group effect.

† Significantly different from the baseline value, *P* < 0.003.

§ Significantly different from Day 7, *P* < 0.003.

### Difference in serum creatinine and eGFR

As shown in Figure 2A, the level of serum creatinine in both groups dropped rapidly after the surgery (Experimental group: 139.2±29.4 μmol/L; Control group: 183.6±198.7μmol/L) and was maintained thereafter. In contrast, the eGFR increased rapidly at day 7 (Experimental group: 57.5±15.2 mL/min; Control group: 65.4±33.2 mL/min) and plateaued through 24 months (Experimental group: 81.0±6.1 mL/min; Control group: 73.1±14.8 mL/min) (Figure 2B). There was no significant difference between the two groups in both serum creatinine (*P* = 0.698) and eGFR (*P* = 0.408).

**Figure 1:**
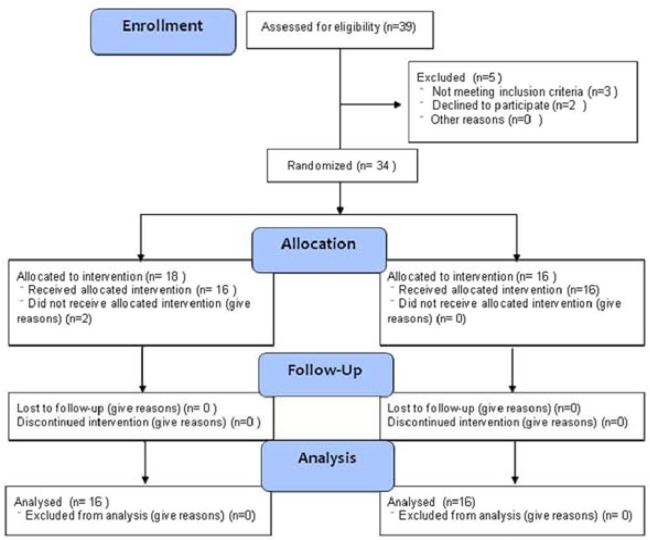
Patient inclusion into the study

**Figure 2:**
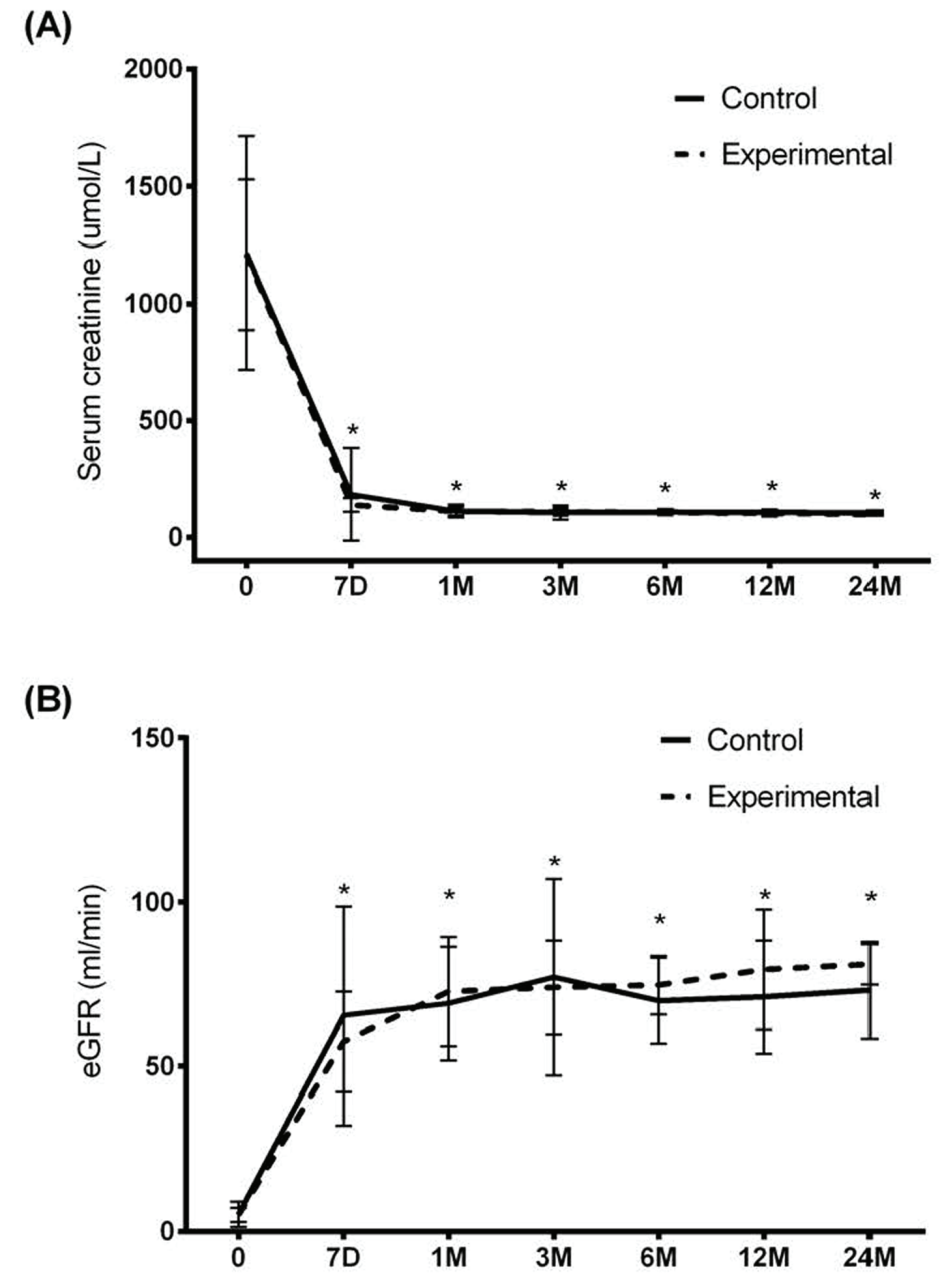
Serum creatinine and eGFR throughout the study period **A.** Serum creatinine and **B.** eGFR were measured in the experimental and control groups. Data were presented as mean (standard deviation). Linear mixed model revealed a significant time effect (*P* < 0.001). Asterisk indicates significantly different from baseline, *P* < 0.003.

### Adverse effects

The percentage of patients with adverse events (AE) in the two groups was similar (*P* = 0.476). As shown in Table 4, lung infection (10 cases) was the most common AE. Other AEs included acute rejection (3 cases in the control group), liver dysfunction (2 cases), anemia (2 cases), perirenal hematoma (1 case), incomplete intestinal obstruction (1 case), diabetes (1 case) and fever (1 case).

**Table 4:** Comparison of the adverse effects between the control (n = 16) and experimental (n = 16) groups

	Control (*n* = 16)	Experimental (*n* = 16)	*P*-value
Acute rejection	3 (18.8)	0 (0.0)	0.226
Diabetes	0 (0.0)	1 (6.3)	1.000
Hyperlipidemia	3 (18.8)	1 (6.3)	0.600
Anemia	0 (0.0)	2 (12.5)	0.484
Lung infection	7 (43.8)	3 (18.8)	0.252
Liver dysfunction	1 (6.3)	1 (6.3)	1.000
*Incomplete intestinal obstruction*	0 (0.0)	1 (6.3)	1.000
Fever	1 (6.3)	0 (0.0)	1.000
Perirenal hematoma	1 (6.3)	0 (0.0)	1.000

Data were presented as count and percentage and tested using Chi-square test.

### Chimerism

The peripheral chimerism of the donor leukocytes was measured at 1, 3 6, 12, and 24 months. No chimerism was detected in any patient.

### Trough concentration of tacrolimus

The trough concentration of tacrolimus was measured from Day 7. Patients treated with low-dose tacrolimus + MSCs had significantly lower trough levels than those who received a standard dose of tacrolimus (*P* < 0.05; Figure S1).

## DISCUSSION

In this pilot study, we compared the outcomes of allograft renal transplant patients treated with low-dose tacrolimus in combination with MSCs with those treated with a standard dosage of tacrolimus. There was no significant difference in acute rejection and graft survival, serum creatinine and eGFR. Although most clinical parameters were similar between the groups, CD38 and NK cells were higher in the control group, suggesting an increased response to cellular stress possibly caused by tacrolimus. Both groups showed similar immune response to donor alloantigens in MLR experiments and the analogous characteristics of T-cell subpopulations measured by flow cytometry.

In addition to its immunosuppressive effects [18], tacrolimus can also cause cytotoxicity [19], including interstitial fibrosis and tubular atrophy, resulting in late dysfunction of transplanted kidneys [20]. Although lower doses of tacrolimus are more desirable to prevent cytotoxicity in renal transplant patients. But, they are associated with a greater risk of acute rejection [21, 22]. Richards et al. [23] recently reported that a tacrolimus trough concentration of ≥8 ng/mL by day 5 was required to prevent biopsy-proven acute rejection. Therefore, other adjunctive therapeutic strategies are required to ensure the safety and efficacy of low-dose tacrolimus. MCSs appear to be a fitting therapeutic target for that purpose, especially given their specific immunosuppressive and immune evasive properties [24].

Autogenic, allogenic, and third party MSCs suppress lymphocyte [15] and leukocyte [12] proliferation. *In vivo* studies have also illustrated their immunosuppressive capacity as intravenous administration of donor MSCs effectively prolonged graft survival in animal models [13, 15]. Specifically, injection of *in vitro*-amplified MSCs into a recipient baboon followed by allogeneic skin graft transplantation showed a delay in transplant rejection and an extension of the graft survival time [12]. Donor-derived MSCs have also induced the long-term acceptance of solid organ allografts [25]. However, the mechanism by which MSCs impart their immunosuppressive effects is not fully known. *In vitro* analyses using co-cultures with transwell assays showed that cell-cell contact was not necessary for suppression [26]. Specifically, MSCs inhibited autologous T cell proliferation through secretion of transforming growth factor-β (TGF-β) and hepatocyte growth factor (HGF) [26]. The low levels of MHC class I and lack of MHC class II, B7-1, B7-2, or CD40 expression by MSCs gives further support that they illicit their immunosuppressive functions through a paracrine factors [24]. Furthermore, Le Blanc et al. [10] showed that the MSC-mediated immunosuppression was dose-dependent and not MHC-restricted, suggesting an important role for MSCs in clinical practice. Although further mechanistic studies are necessary to fully determine the role of MSCs in allo-rejection, their use in clinical trials is increasing [24].

Perico et al. [27] showed that autologous MSCs prevented acute rejection in two renal transplant patients who received kidneys from living-related donors and received maintenance immunosuppression with cyclosporine and mycophenolate mofetil. In a phase I clinical study that included six renal transplant patients, Reinders et al. [28] found that autologous bone marrow-derived MSCs provided systemic immunosuppression and was safe at 1-2×10^6^ cells per kilogram of body weight. Reinders' group has subsequently initiated a phase II study that will include 70 renal allograft recipients to examine if MSCs with everolimus, a mammalian target of rapamycin (mTOR) inhibitor, can facilitate tacrolimus withdrawal and decrease fibrosis as well as opportunistic infection [29]. Similarly, Tan et al. [16] showed the efficacy of autologous MSCs even at a 20% reduction in the dosage of calcineurin inhibitors.

We chose the combination of low-dose tacrolimus with MSCs rather than a tacrolimus withdrawal due to the toxicity of MSCs observed with high doses of tacrolimus reported by Hoogduijn et al. [30]. Furthermore, increased immunosuppression by MSCs was observed following preincubation with tacrolimus, which is suggestive of their additive potential [30]. Our results indicate that donor-derived MSCs combined with low-dose tacrolimus has similar efficacy to standard immunosuppression following living-related renal transplantation. Given that a randomized trial of 74 patients showed a significant reduction in acute GVHD with tacrolimus and sirolimus, a mTOR inhibitor, compared with tacrolimus and methotrexate [31], further studies will analyze the effect of including MSCs with tacrolimus and sirolimus.

Although previous studies have shown immunosuppressive activities for MSCs and suppression of graft-*versus*-host disease [32, 33], which is in agreement with the results of the present study, further studies are necessary to define the exact protocol for the culture, expansion, and administration of MSCs [34]. In rats, MSCs injected into the renal artery were retained in the glomeruli, lowering the frequency of glomerulonephritis and preventing acute cellular rejection [35, 36]. However, our studies are the first to show that the administration of MSCs by intra-arterial injection is feasible and safe as was evidenced by the absence of embolism, thrombosis, infection, or any other complications, within 12 months of transplantation. We hypothesize that direct injection of MSCs into the renal artery may locally depress the inflammatory response and, therefore nonspecifically protect the graft.

Donor leukocytes can be detected many years after solid organ transplantation in recipients with long-term graft survival and who were able to safely reduce or discontinue immunosuppression therapy [37]. In the present study, chimerism was undetectable at 3 and 12 months post-transplantation. In addition, recent studies of transplant patients who maintain stable kidney graft function in the absence of immunosuppression drugs showed that these patients have more peripheral B cells [38, 39]. In the present study, patients in the experimental group had more peripheral B cells than those in the control group at 3 months. However, further research is required to determine whether the variation of peripheral B cells alters long-term graft function.

In addition to the small study sample, the present study is limited in the lack of mechanistic data. In addition, although the immunosuppressive properties of MSCs have been well-established, MSC are still immunogenic to a certain extent [24], which may limit their clinical application. Finally, MSC therapy may also be limited in that their effects do not persist following infusion, necessitating further applications.

In conclusion, immunosuppression can be maintained after allograft renal transplantation with low-dose tacrolimus (∼60% of the standard dosage) in combination with donor-derived MSCs, which may decrease the nephrotoxicity associated with many immunosuppressants. Further research is needed to determine the minimum effective dosage of tacrolimus when used in combination with MSCs. Further mechanistic analyses of the combination therapy are also required. The findings could be beneficial in ensuring long-term graft survival after renal transplantation.

## MATERIALS AND METHODS

### Patient characteristics

The kidney transplantations were performed from September 2009 to January 2011. As shown in Figure 1, 39 patients were assessed for eligibility. Three did not meet the inclusion criteria, and two declined to participate. Therefore, 34 uremia patients were assigned to receive either experimental or control intervention, but two patients did not. The remaining 32 patients were divided into an experimental group (MSC group, *n* = 16) and a control group (non-MSC group, *n* = 16).

### Study design

The materials and methods of this study are similar to those of our pilot study. They were outlined in our previous publication and are restated here in their entirety, except for changes that are pertinent to the follow-up study [40]. Donors and recipients undergoing living-related kidney transplantation procedures in the Second Affiliated Hospital of Guangzhou Medical University were considered for enrollment in this prospective, nonrandomized pilot study. Donor selection complied with the 2004 Amsterdam Forum Guidelines [41] and the 2007 Chinese “Regulation on Human Organ Transplantation” (Order of the State Council No. 491) [17]. All candidates met the following inclusion criteria: (a) patients were undergoing primary kidney transplantation; (b) donors and recipients were 18 to 60 y of age and ABO compatible; (c) the primary kidney disease was chronic glomerulonephritis; (d) tacrolimus, rather than cyclosporine A, was used as the maintenance immunosuppressant; and (e) complement-dependent cytotoxicity examination and panel reactive antibody examination were negative (G10%) before kidney transplantation.

Potential enrollees were excluded if (a) kidney transplantation was secondary, multiple, or combined with the transplantation of other allograft organs; (b) recipients had systemic or active infections; (c) recipients had a history of severe cardiovascular or pulmonary dysfunction, malignancy, liver dysfunction, and chronic enteritis; (d) recipients had diabetes mellitus or other glycometabolic disorders; and (e) tacrolimus had to be replaced with another immunosuppressant(s) after kidney transplantation. The participants were consecutive candidates for transplantation that were eligible for the study and were assigned into the experimental group or the control group at their choice.

As induction therapy, all enrolled recipients were prescribed Cytoxan (200 mg/day) and methylprednisolone (750, 500, and 250 mg/day) from days 0 to 3. Beginning on day 4, patients in the control group received a standard dose of tacrolimus (0.07-0.08 mg/kg/day), whereas patients in the experimental group received a low dose of tacrolimus (0.04-0.05 mg/kg/day) and two infusions of MSCs. The first infusion of MSCs (5×10^6^ cells) was delivered directly into the renal allograft artery at the time of kidney transplantation. Briefly, a Gibson's incision was made at the lower abdomen, and the internal iliac artery and external iliac vein were exposed. End-to-end anastomosis was performed between the renal and internal iliac arteries, and end-to-side anastomosis was performed between the renal and the external iliac veins. Prior to finishing the last stitch during the arterial anastomosis, a 22F catheter needle was inserted from the anastomosis site along the direction of blood flow, fixed, and connected to a sterile transfusion apparatus. When restoring the blood flow to the transplanted kidney, 10 mL of saline-diluted MSCs (5×10^6^) were quickly infused (within 2 min) using a pressurizer. The catheter was removed after infusion, and the gap was sutured. Inverted mastoid-shaped anastomosis was carried out between the ureter and the bladder. After one month, the second infusion (2×10^6^ cells/kg diluted in 50 mL normal saline) was administered intravenously over 20 min. Mycophenolate mofetil (1 g/day) and prednisone were also prescribed to patients in both groups. An oral administration of prednisone was initiated at 30 mg/day at day 4 after kidney transplantation and then tapered by 5 mg every week to the maintenance dose of 15 mg/day.

Patients were followed up for at least 2 y (the original 12 patients were followed up for up to 4 y) after kidney transplantation. The 2-y cumulative rates of acute rejection in both groups were compared. Acute rejection was diagnosed based on clinical manifestations. Serum creatinine was examined at baseline, and at 7 days as well as 1, 3, 6, 12 and 24 months after treatment.

This study was performed in accordance with the Declaration of Helsinki and was approved by the Ethical Committee of the Second Affiliated Hospital of Guangzhou Medical University. Written informed consent was obtained from all recipients and donors.

### MSC isolation and characterization

MSC isolation and identification was undertaken at the Center for Stem Cell Biology and Tissue Engineering of Sun Yat-sen University. Briefly, approximately 50 mL of bone marrow aspirate from healthy adults was diluted in 100 mL of PBS after which separation medium (Sigma-Aldrich, St. Louis, MO, USA) was added at a ratio of 1:2 to form a clear interface. After centrifugation at 2000 rpm for 20 min, the mononuclear cells were extracted, transferred to a new centrifuge tube, mixed with PBS (1:5, *v*:*v*), and centrifuged at 2000 rpm for 10 min. After the cells were washed with 10 mL PBS, the supernatant was removed, 4 mL of low-glucose medium (GIBCO-Life Technologies, Thermo Fisher Scientific, Inc. Waltham, MA, USA) was added, and the cells were cultured in a 37°C incubator. The low-glucose medium was changed once every three days. After approximately 10 days, cell colonies were formed, and 0.25% trypsin was used for cell dissociation. The cells were sub-cultured at a ratio of 1:2 until reaching a total of 10^8^ cells over five passages after which they were preserved in liquid nitrogen (see Figure S1A, SDC, http://links.lww.com/TP/A728).

Flow cytometry analysis of 1×10^5^ cells (in 100 μL) incubated with 10 μL of fluorophore-labeled antibodies (1:1000) revealed that the isolated cells expressed the surface markers, CD29, CD44, CD73, CD90, CD105, and CD166, but not the hematopoietic markers, CD45 and CD34 (see Figure S1B, SDC, http://links.lww.com/TP/A728). All antibodies and isotype-matched controls were from eBioscience (San Diego, CA). Cells were acquired using a multicolor cytometer MoFlo Astrios (Beckman Coulter, Brea, CA, USA), and data were analyzed with CellQuest Pro software (Becton Dickinson, Franklin Lakes, NJ).

After the sixth passage, the multiple differentiation capacity of MSCs was confirmed by 0.5% oil red staining for adipogenic differentiation (see Figure S1C, SDC, http://links.lww.com/TP/A728) and 0.1% alizarin red staining for osteogenic differentiation (see Figure S1D, SDC, http://links.lww.com/TP/A728) as described previously [42, 43].

### Immune monitoring

White blood cells were routinely counted before transplantation (day 0) and at 3, 6, and 12 months after transplantation. Immunophenotyping for characteristics of T lymphocytes and related subpopulations (CD3^+^CD4^+^CD8^−^ and CD3^+^CD4^−^CD8^+^), total B lymphocytes, and NK cells (CD56^+^CD3^−^NKG2A/NKG2D) was performed using 100 μL of whole blood with 20 μg of antibody with a multicolor flow cytometry (all antibodies and isotype-matched controls were from eBioscience). Cells were acquired using a multicolor cytometer MoFlo Astrios (Beckman Coulter), and data were analyzed with CellQuest Pro software (Becton Dickinson).

### Mixed lymphocytes reaction (MLR)

The one-way MLR was conducted to evaluate the recipients' response to donor alloantigen challenge. Peripheral blood mononucleated cells (PBMCs) were obtained (and frozen) from 12 recipients at day 0 and months 3, 6, and 12 and labeled with carboxyfluorescein diacetate, succinimidyl ester (Sigma-Aldrich, St. Louis, MO, USA). Aliquots of 1×10^5^ recipient PBMCs were plated and stimulated with 1×10^5^ donor PBMCs pretreated with mitomycin C (Sigma). Cultured cells were harvested after 6 days, and proliferation was measured by FACSCalibur flow cytometer (Becton Dickinson).

### Detection of chimerism

Chimerism was assessed at 3 and 12 months. PCR co-amplification of 16 euchromosomal short-tandem repeat loci (D8S1179, D21S11, D7S820, CSF1PO, D3S1358, THO1, D13S317, D16S539, D2S1338, 19S433, vWA, TPOX, D18S51, D5S818, FGA, and AMEL) was performed in a fluorescence-based multiplex reaction using the AmpFLSTR Identifier kit (Applied Biosystems, Foster City, CA, USA). All loci were amplified using a GeneAmp PCR System 9600 (Applied Biosystems). The amplified products were detected by capillary electrophoresis using an ABI 3130XL DNA Genetic Analyzer (Applied Biosystems). Short-tandem repeat profiles were analyzed using GeneScan and Genotyper Analysis Software (Applied Biosystems).

### Determination of estimated glomerular filtration rate (eGFR)

The eGFR (i.e., flow rate of filtered fluid through the kidney) was determined using a Discovery VH (GE Healthcare, LLC, Wauwatosa, WI, USA). eGFR was calculated using the follow formula: eGFR (mL/min/1.73 m^2^) = 186 × (creatinine, mg/dL)^−1.154^ × (age, y)^−0.203^ (× 0.742 if female).

### Statistical analysis

Normally distributed interval data were presented as mean (standard deviation, SD) and tested by independent t-tests. Median and inter-quartile ranges were calculated for interval data with skewed distributions, and Mann-Whitney *U* tests were further applied to test the differences between control and experimental groups. Categorical variables were expressed as count (percentage) and tested using Chi-square tests; however, if there were more than 20% of cells with expected value less than five cells, Fisher's exact tests were applied instead. Linear mixed models encompassed fixed effects of time and group and were implemented for longitudinal data with normal distributions. If longitudinal data with skewed distributions existed, Friedman tests and Mann-Whitney *U* tests were used instead to test the time effect and group effect, respectively. A two-tailed alpha level was set at 0.05. The alpha level was adjusted to 0.003 when multiple comparisons were applied. Statistical analyses were assessed using SPSS software version 15.0 (SPSS Inc, Chicago, IL, USA).

## SUPPLEMENTARY MATERIAL FIGURE


