# Supplementary material for: Low-dose tacrolimus combined with donor-derived mesenchymal stem cells after renal transplantation: a prospective, non-randomized study
Source: Oncotarget. 2016 Feb 25;7(11):12089–101. doi: 10.18632/oncotarget.7725 (PMC4914271; doi:10.18632/oncotarget.7725)
Supplement: Supplementary file 1 [file oncotarget-07-12089-s001.pdf]

Low-dose tacrolimus combined with donor-derived mesenchymal stem cells after renal transplantation: a prospective, non-randomized study

Supplementary Material

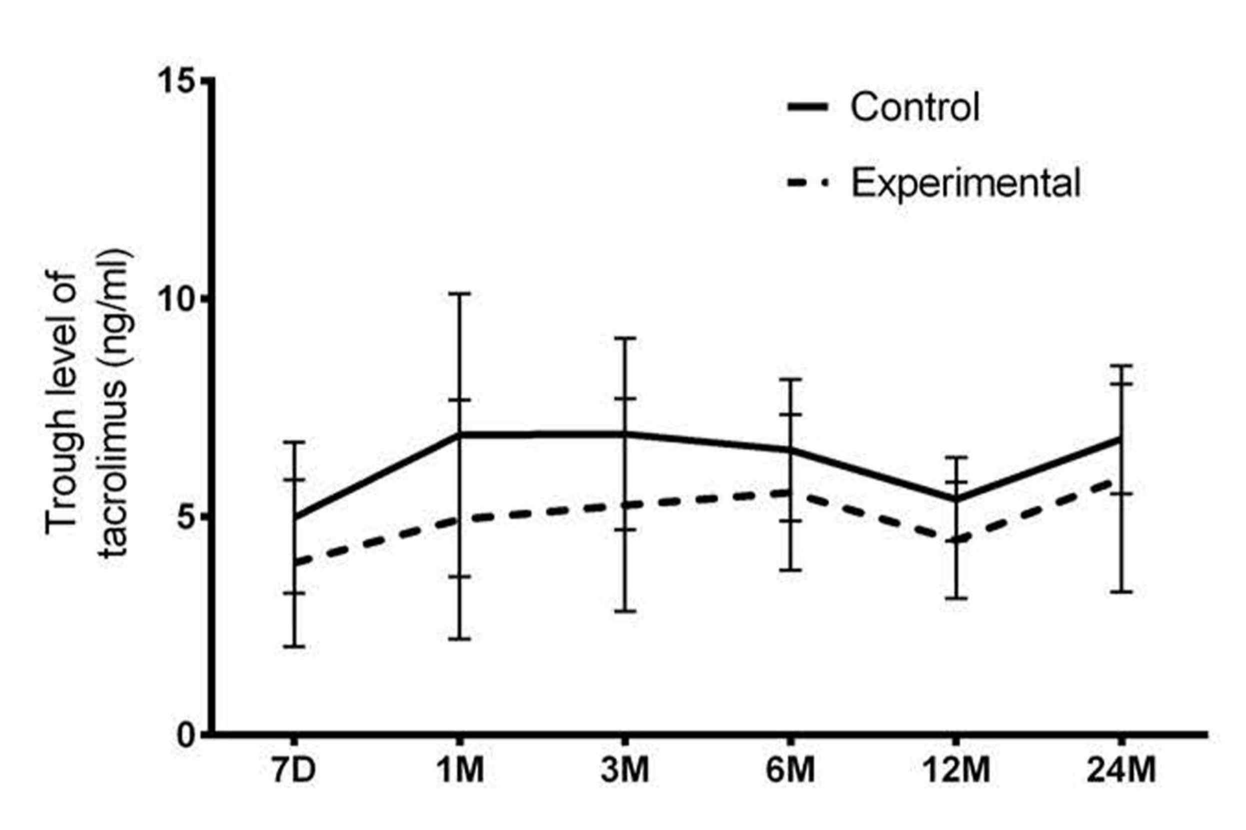

Supplementary Figure S1: Trough level of tacrolimus over time in 32 patients.
